# Effective Data Collection Approaches for Citizen Science in Biodiversity Research

**DOI:** 10.1002/ece3.73461

**Published:** 2026-04-08

**Authors:** Olena Kozak, Aino Erkinaro, Kaisa‐Leena Huttunen, Larysa Osypenko, Svitlana Kyiak, Nataliia Matsai, Mykyta Peregrym

**Affiliations:** ^1^ National University of “Kyiv‐Mohyla Academy” Kyiv Ukraine; ^2^ University of Oulu Oulu Finland; ^3^ Finnish Environment Institute Oulu Finland; ^4^ Luhansk Taras Shevchenko National University Myrhorod Ukraine; ^5^ Nicolaus Copernicus University in Toruń Toruń Poland

**Keywords:** data collection, digital platforms, GBIF, public engagement, social media, species observations

## Abstract

Biodiversity underpins the stability of ecosystems and human well‐being. However, it is rapidly declining globally, necessitating robust and scalable monitoring solutions. Citizen science has emerged as a powerful complement to traditional biodiversity research, enabling the collection of widespread data and fostering public engagement, particularly through digital platforms such as iNaturalist and eBird. This study investigates global patterns in citizen science contributions to biodiversity monitoring, exploring the influence of socioeconomic factors, national biodiversity value, and platform‐specific contributions on data generation. Key strategies for optimizing public participation, enhancing data quality and coverage are identified through statistical analysis and three in‐depth case studies from Finland and Ukraine. Our findings show that citizen science platforms now provide a significant portion of biodiversity records in GBIF. Citizen science contributions to global biodiversity data are shaped more by a country's ecological uniqueness and biodiversity value than by its socioeconomic development, as effectiveness depends not just on user numbers or observations but also on biodiversity significance, platform origin, and user engagement. Combining digital platforms with targeted outreach, through social media, personal communication, and expert validation, emerges as a promising strategy to enhance data reliability and participant engagement. This mixed‐methods approach proves especially effective in contexts with varying levels of digital access, biodiversity richness, and species monitoring needs.

## Graphical abstract

This study examines global patterns in citizen science contributions to biodiversity monitoring, investigating the impact of socioeconomic factors, national biodiversity value, and platform‐specific effectiveness on data generation. Key strategies for optimizing public participation and enhancing data quality and coverage are identified through statistical analysis and three in‐depth case studies from Finland and Ukraine.

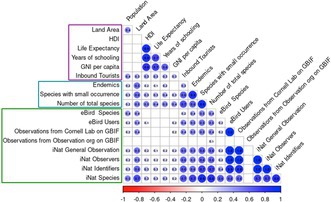

## 1 Introduction

Biodiversity is essential for sustaining life on Earth, and its rapid decline seriously threatens human well‐being (Reid et al. 2005; UN 1992; Díaz et al. 2015; Isbell et al. 2017). Effective monitoring is therefore a keystone of biodiversity research and conservation (CBD 2022). In this context, citizen scientists and volunteers (Cambridge Dictionary 2025) play an increasingly vital role, contributing valuable data on species occurrence, abundance, and phenology (Chandler et al. 2017; CBD 2020; Bowler et al. 2022). In Europe, for instance, 87% of individuals involved in species monitoring are volunteers (Schmeller et al. 2009), and over 60% of newly described animal species since the 1950s have been discovered or rediscovered by nonprofessional taxonomists (Fontaine et al. 2012).

Citizen science helps address critical data gaps from traditional monitoring systems, enriching official datasets and improving forecasting accuracy. At the same time, it fosters public engagement, environmental awareness, and a sense of stewardship, making it a key component in advancing global policy frameworks such as the Sustainable Development Goals, the Convention on Biological Diversity, and related initiatives (CBD 2020; Bowler et al. 2022; EPA Network 2022; Danielsen et al. 2024). Notably, out of the 365 indicators in the Kunming–Montreal Global Biodiversity Framework, 110 (30%) can be directly incorporated into citizen science, while 185 (51%) stand to benefit from public participation in data collection (Danielsen et al. 2024). Additionally, citizen science provides a flexible and resilient way to monitor biodiversity, especially in situations where resources are limited or during periods of war, crisis, or other emergencies (Kozak et al. 2024).

Citizen science offers mutual benefits: while contributing valuable data to biodiversity research, citizen scientists gain various personal rewards. These include increased self‐efficacy, knowledge, a deeper connection to nature, a sense of community, and different physical and mental health benefits (Peter et al. 2019; Dimson and Gillespie 2023; Eichholtzer et al. 2024). Maintaining direct human contact with natural ecosystems is more important in an era of rapid digitalization and shifting socioecological relationships.

At the same time, digitalization and the advancement of Artificial Intelligence (AI) are accelerating the generation of biodiversity data (Vohland et al. 2021; Strasser et al. 2023), while also presenting significant challenges in ensuring data quality and validation (Lotfian et al. 2021; López‐Guillén et al. 2024). For example, iNaturalist (2025), one of the most widely used citizen science tools, offers numerous advantages, such as ease of use, low technical requirements, real‐time sharing, open access, community engagement, AI‐assisted identification, and automatic integration with the Global Biodiversity Information Facility (GBIF 2025), yet it also faces challenges including low‐quality images, identification errors, duplicate reports, and limited geographic accuracy, all of which can impact the reliability and utility of its records for species research and conservation (Dimson and Gillespie 2023; López‐Guillén et al. 2024). While herbarium specimens can contain errors, misidentifications are more common in iNaturalist Research Grade observations (White et al. 2023). Only about 30%–59% of lichen species on iNaturalist with Research Grade observations are correctly identified (McMullin and Allen 2022; Munzi et al. 2023).

The number of people with detailed local biodiversity knowledge who avoid digital platforms due to personal beliefs, limited internet access, or low digital literacy is unknown, but could be potentially high. This raises the question of which approach of citizen science, modern AI applications, or traditional methods like personal interviews is most effective and reliable, and whether they are equally suited to the same purposes.

This paper aimed to explore global patterns in citizen science contributions to biodiversity data collection, investigating how socioeconomic factors and biodiversity value influence data generation, identifying the most effective citizen science platforms, species data collection methods, and public engagement strategies. Main trends in citizen science development, key strategies for optimizing public participation, enhancing data quality and coverage are identified through analysis of global statistics and three in‐depth case studies from Finland and Ukraine. To achieve this aim, the following research questions are addressed:

- How do socioeconomic factors and the biodiversity value (uniqueness) of different countries influence the volume of data contributed to the GBIF through citizen science?
- Which citizen science platforms contribute most significantly to data generation in the GBIF, and how effective are they?
- What are the most effective methods and channels for engaging the public in species observation and data collection through citizen science, based on evidence from three case studies?
- Do the most effective methods and channels for citizen science differ depending on the purpose and species status, based on evidence from three case studies?

## 2 Materials and Methods

In this paper, we analyzed statistical data to explore global patterns and assess how socioeconomic factors and the biodiversity value of countries worldwide influence the volume of data contributed to the GBIF through citizen science. We also examined which citizen science platforms contribute the most and assessed their effectiveness. Additionally, we present three case studies from Finland and Ukraine that highlight how citizen scientists collect data on various species, helping to identify the most effective channels for recording species observations and engaging public participation.

### 2.1 Analysis of Socioeconomic and Biodiversity Factors

To assess the influence of socioeconomic and biodiversity factors on citizen science data generation, two types of data were collected for all countries worldwide: citizen science indicators and socioeconomic and biodiversity indicators. Citizen science indicators include the number of species recorded on eBird (eBird 2025), the number of eBird users (eBird 2025), occurrences on GBIF from the Cornell Lab of Ornithology (representing eBird data) (GBIF 2025), occurrences on GBIF from Observation.org (GBIF 2025; Observation.org 2025), as well as the number of observations, observers, identifiers, and recorded species (all taxa, plant, and bird) on iNaturalist (iNaturalist 2025). Citizen science data were manually exported from all three sources across all categories, including records for the entire period up to February 2025, and compiled into a single Excel file for further analysis. The socioeconomic indicators include the number of inbound tourists in 2019 (UN Tourism 2025), land area (World Bank Group 2025a), population (World Bank Group 2022), and key development metrics such as the Human Development Index, GNI per capita, years of schooling, and life expectancy (UNDP 2025). Biodiversity indicators include the total number of species, the number of endemic species, and the number of species with limited occurrences (World Bank Group 2025b). All data were collected into a single file and analyzed for correlation by generating a correlation matrix in the R software environment. Correlations were considered moderate when the coefficient was between 0.5 and 0.7 (0.5 < *r* ≤ 0.7), and strong when it exceeded 0.7 (*r* > 0.7) (Hazra and Gogtay 2016; Akoglu 2018; numiqo Team 2026). The limitation of the study is that correlation analysis assumes linear pairwise relationships and does not account for potential nonlinear effects or multivariate interactions among the analyzed variables. Since this is an exploratory study, more detailed and in‐depth statistical methods were not used, but it is only the first step, and the detailed analysis will follow.

Data from the GBIF website (GBIF 2025) were analyzed to assess the dynamics of occurrences, top publishers, citation rates, and other main statistics available on the GBIF platform, which are important for revealing main trends in platform development.

The processed data were analyzed and visualized in Excel, with an exception correlation plot generated using an R script in Posit Cloud. All supplementary files and scripts are available via the link—https://anonymous.4open.science/r/CitSci‐in‐World‐4D71.

### 2.2 Case Study 1: Marmot and Hamster Population Data in the Sumy and Poltava Regions (Ukraine)

The marmot ( *Marmota bobak* (Müller, 1776)) and the European hamster ( *Cricetus cricetus* (Linnaeus, 1758)) are listed in the Red Data Book of Ukraine (Order… 2021a). *C. cricetus* is also included in Annex II of the Bern Convention (Council of Europe 1976). Population data for both species were collected between April 16 and July 24, 2024 in the Sumy and Poltava regions of Ukraine. By the 1940s, marmots in Europe had sharply declined, surviving only in isolated farmland and reserves, but reintroductions and natural recolonization began from the 1980s (Grubnyk and Tokarsky 2020).

To gather data about marmot and hamster localities, the questionnaire was distributed through local Facebook groups, official webpages (Poltava Regional Department of Ecology; Luhansk Taras Shevchenko National University), and local print media (Table S1). Additional data were gathered through a literature review (Merzlykin et al. 1996) and personal interviews. In total, 85 respondents participated in the study. The species' locality refers to a broader area near a specific settlement, with coordinates, an encounter description, and an approximate location. Its accuracy is verified through satellite imagery analysis and field surveys. One response was excluded from the table because the species could not be identified. All responses were carefully analyzed, and new localities were evaluated to avoid duplication. The research results are presented in Table 1.

**TABLE 1:** Results of citizen science involvement in marmot ( *Marmota bobak* ) and hamster ( *Cricetus cricetus* ) localities research in Sumy and Poltava Regions, Ukraine.

Data category	*Marmota bobak*	*Cricetus cricetus*
Total number of species localities reported by citizen scientists	95	3
Through personal interviews	43	1
Through local Facebook groups	25	2
Through the official Facebook page	3	0
Through the Internet and print media	4	0
Through local amateur communities	13	0
Other	7	0
Number of new localities identified through citizen scientist reports	40	3
Species localities documented in research papers (Merzlykin et al. 1996)	11	—
Number of species localities recorded in iNaturalist, GBIF, and UkrBin before the start of the research	23	2

### 2.3 Case Study 2: Citizen Science Contributions to Non‐Native Pink Salmon Research in Finland

The Pacific pink salmon *Oncorhynchus gorbuscha* (Walbaum, 1792), also known as humpback salmon, a non‐native semelparous salmonid species, where all adults die after a single spawning event, has in recent years shown significant influxes into the northernmost rivers of Northern Europe, where it was previously encountered only occasionally (ICES 2022, 2024; Diaz Pauli and Utne 2024). Data on pink salmon carcass sites were collected from the end of July to the middle of September 2023, along the river Teno (a.k.a. Tana/Deatnu) watershed in northern Finland and Norway. The main goal of the use of citizen science was to collect data on the habitat types where pink salmon carcasses end up. Data were collected by publishing ads in print media, social media (Facebook groups, X), and printed ads in storefronts, asking local people and tourists to report sites of pink salmon carcasses (Table S1). Reporting was done by sending a text to a given phone number. Participants were informed about the processing of personal data. All messages were deleted after the coordinates were saved, ensuring that personal information, such as names or phone numbers, was not recorded or stored in any register. Additional data were gathered through personal communication (e.g., locals talking to the researchers while grocery shopping). In total, 22 reports were received (Table 2). The reports included information about the location, number of observed carcasses, approximate descriptions of the environment (e.g., stream depth and particle size), and, in some cases, pictures of carcass accumulation sites. Based on the information, most of these sites were visited by the research team, and environmental variables (depth, velocity, particle size) were measured individually from all carcass spots within a site.

**TABLE 2:** Results of citizen science involvement in non‐native pink salmon ( *Oncorhynchus gorbuscha* ) research in Finland.

Data category	Number of carcass/locality reports received	Number of individual spots measured based on the reports
Total number of species localities reported by citizen scientists	22	188
Through personal communication	5	24
Through social media	3	23
Through text messages	14	141
Number of species localities recorded on iNaturalist	3	—
Number of species localities recorded on LAJI	12	—
Number of localities personally discovered by the author	6	60

### 2.4 Case Study 3: Plant Species Data in the Dnipropetrovsk Region (Ukraine)

Field research with the involvement of citizen scientists was conducted from 2020 to 2024 in the Dnipropetrovsk region of Ukraine. The study focused on four categories of plant species:

1. Rare species listed in the Red Data Book of Ukraine (Order… 2021b): *Astragalus ponticus* Pall., *Adоnis vernаlis* L., *Gladiolus tenuis* M.Bieb, *Anacamptis morio* (L.) R.M. Bateman, Pridgeon & M.W. Chase;
2. Regionally rare species (Official lists… 2012): *Asplenium trichomanes* L., *Ephedra distachya* L., *Onosma tinctoria* M.Bieb., *Sempervivum ruthenicum* Schnіttsp. & C.B. Lehm;
3. Narrowly distributed native species in the study area (Kucherevsky 2001; Travleev 2010; Tarasov 2012): *Dianthus trifasciculatus* Kit. ex Schult., *Dianthus carbonatus* Klokov, *Centaurium erythraea* Rafn., *Jurinea cyanoides* (L.) Rchb.;
4. Alien and invasive species (Protopopova and Shevera 2019; Mosyakin and Mosyakin 2021): *Asclepias syriaca* L., *Grindelia squarrosa* (Pursh) Dunal, *Datura* spp.

The plant species names are provided following the accepted nomenclature from the open‐access database Plants of the World Online (POWO 2025). It should be noted that this research refers to *Datura* spp., due to possible confusion in species identification. However, *Datura stramonium* L. is the most widespread and predominant species in the study area.

Citizen scientist participation and communication were facilitated through Facebook (2025) posts, printed publications, and direct interactions with local people during field expeditions. Species occurrence data were categorized based on their source, including submissions from citizen scientists via phone calls, emails, personal conversations, and Facebook. Upon receiving a species locality report, we personally visited the sites, conducted on‐site examinations, took photographs, and occasionally collected herbarium specimens. These verified records were then added to iNaturalist. None of the citizen scientists requested credit as the discoverer or claimed authorship of their findings. The number of species localities on iNaturalist also includes the author's verified data. Additionally, the dataset consists of field data collected directly by Svitlana Kyiak during expeditions (Kyiak 2024a, 2024b), along with supplementary data from scientific literature sources (Kucherevsky 2001; Travleev 2010; Tarasov 2012) and mixed publications authored by both researchers and amateur biologists (UNCG 2019, 2020, 2023a, 2023b, 2024). The research results are presented in Table 3.

**TABLE 3:** Outcomes of citizen science participation in floristic research in the Dnipropetrovsk Region, Ukraine.

Data category	Number of localities documented in the scientific literature (Kucherevsky 2001; Tarasov 2012; Travleev 2010)	Number of localities documented in other literature (UNCG 2019, 2020, 2023a, 2023b, 2024)	Number of localities personally discovered by the author	Number of localities documented on iNaturalist	Number of locations discovered with the assistance of citizen scientists				
					During a personal interview	Via Facebook	By phone	By email	Total
Red Book of Ukraine									
*Astragalus ponticus* Pall.	9	14	4	26	2	3	1	0	**6**
*Adоnis vernаlis* L.	9	70	2	22	1	0	0	0	**1**
*Gladiolus tenuis* M.Bieb	12	2	0	8	1	0	0	0	**1**
*Anacamptis morio* (L.) R.M. Bateman, Pridgeon et M.W. Chase	4	0	1	1	0	0	0	0	**0**
Regional rare species									
*Asplenium trichomanes* L.	8	2	1	6	0	0	0	0	**0**
*Ephedra distachya* L.	≥ 20	1	10	25	2	2	1	0	**5**
*Onosma tinctoria* M.Bieb.	6	1	6	10	0	0	0	1	**1**
*Sempervivum ruthenicum* Schnіttsp. Et C.B. Lehm	15	2	1	39	0	0	0	0	**0**
Narrowly distributed native species in the study area									
*Dianthus trifasciculatus* Kit. ex Schult.	1	—	7	9	1	0	2	0	**3**
*Dianthus carbonatus* Klokov	10	—	2	6	0	0	1	0	**1**
*Centaurium erythraea* Rafn.	7	—	1	1	0	0	1	0	**1**
*Jurinea cyanoides* (L.) Rchb.	14	—	5	19	1	0	1	0	**2**
Alien and invasive species									
*Asclepias syriaca* L.	18	3	3	73	3	10	3	1	**17**
*Grindelia squarrosa* (Pursh) Dunal	5	95	1	37	2	2	1	0	**5**
*Datura* spp.	3	0	2	17	0	0	1	0	**1**

## 3 Results

### 3.1 Influence of Socioeconomic and Biodiversity Factors on Citizen Science Data Contribution

**FIGURE 1:**
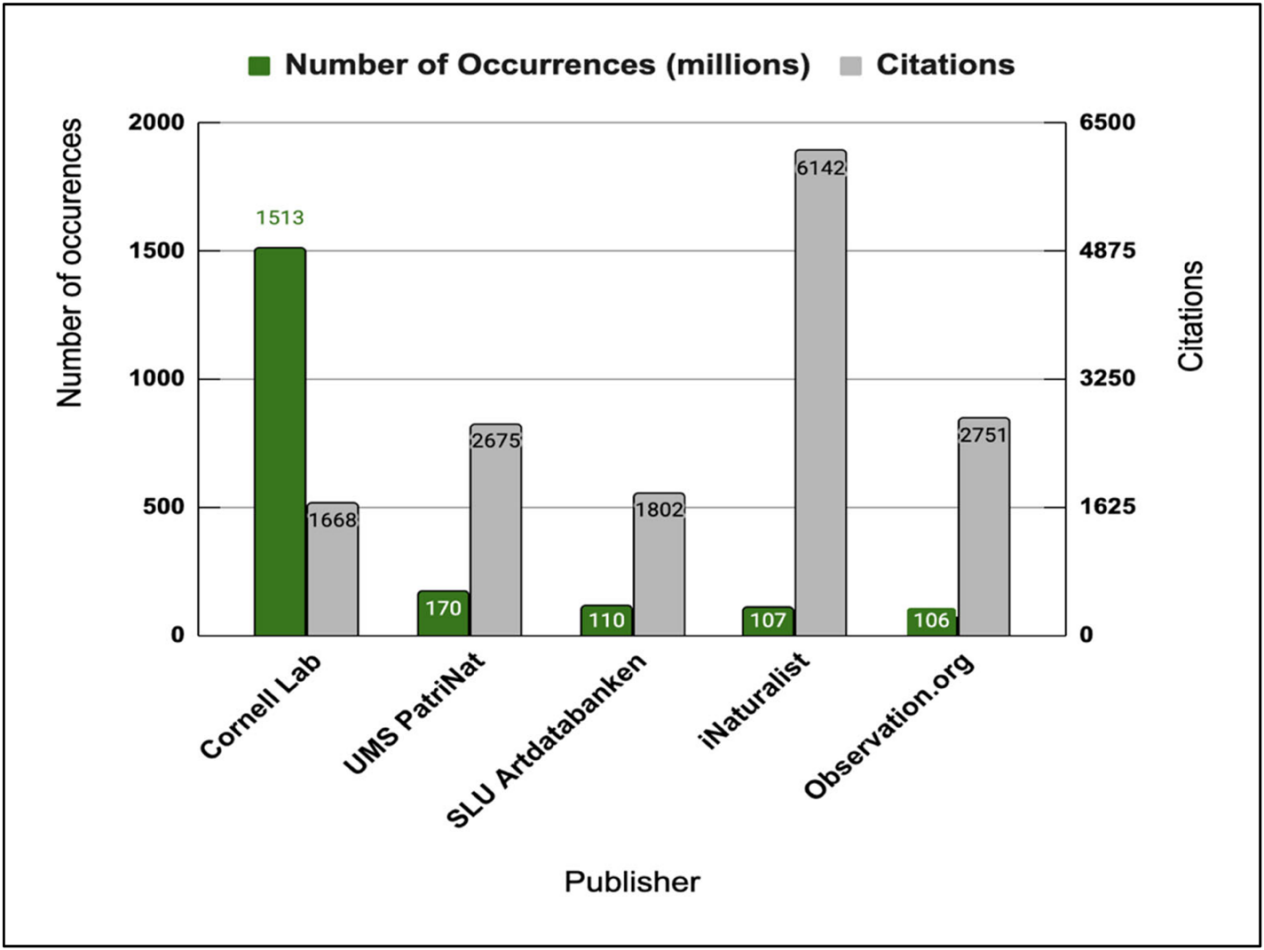
Top five publishers on GBIF by the number of occurrences, with citation rates included (Data Source: GBIF 2025).

The world's largest biodiversity database is GBIF (2025). The top five publishers on GBIF include The Cornell Lab of Ornithology (eBird), UMS PatriNat, SLU Artdatabanken, iNaturalist, and Observation.org (Figure 1). Three (eBird, iNaturalist, and Observation.org) of these five publishers are fully citizen science platforms. Notably, among the top publishers, the most cited datasets come from iNaturalist and Observation.org (Figure 1).

**FIGURE 2:**
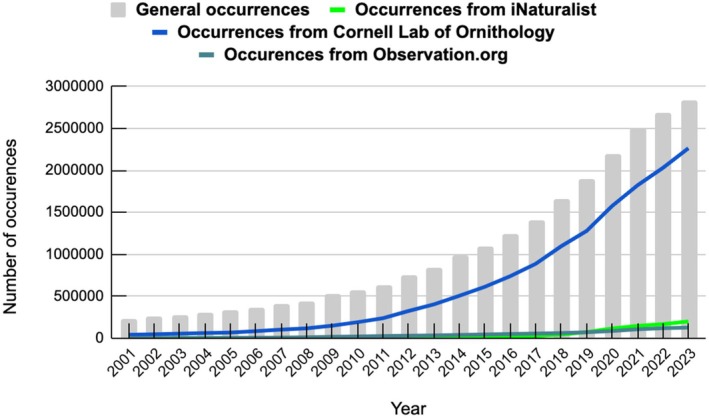
General occurrences on GBIF to occurrences from Cornell Lab of Ornithology (eBird), iNaturalist, and Observation.org from 2001 to 2023 (Data Source: GBIF 2025).

The eBird is one of the world's most popular citizen science platforms. Since beginning to publish data to GBIF in 2013, it has become the platform's largest data contributor. Other major contributors include iNaturalist.org, which became a GBIF publisher in 2012, and Observation.org, which has been a GBIF publisher since 2018 (Figure 1). Observations collected before the data publishing start date from these three sources have been retroactively added to GBIF (GBIF 2025). The percentage of occurrences from the eBird on GBIF has steadily risen from 18% in 2001 to 80% in 2023 (Figure 2). Similarly, occurrences from iNaturalist have increased, though to a lesser extent, growing from 1% in 2010 to 7% in 2023. A comparable trend is observed for Observation.org, with its share rising from 0.4% to 4.4% over the same period. The eBird has been the leading GBIF publisher since 2001, while iNaturalist became the second largest in 2020, and Observation.org has ranked third since 2021. This indicates that data collected through citizen science tools dominate at the GBIF, and the platform's data volume has grown due to citizen science contributions. At the same time, our data (Figure 1) show that the eBird is cited less frequently than iNaturalist. This may be because eBird collects data only on birds, while iNaturalist and Observation.org cover all taxonomic groups.

**FIGURE 3:**
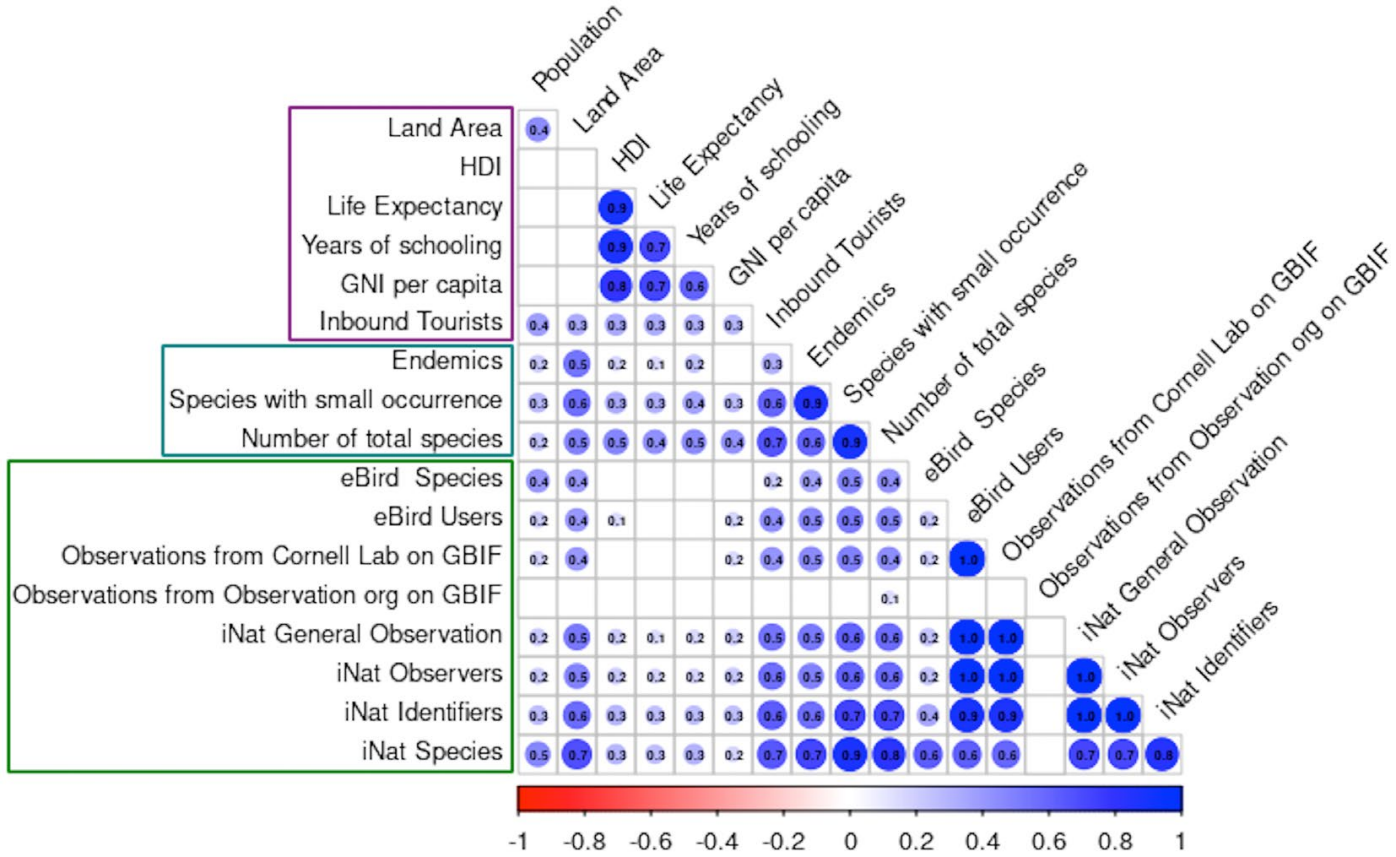
Correlation plot illustrating relationships among socioeconomic factors (in a pink frame), biodiversity values (in a teal frame), and citizen science indicators (in a green frame); nonsignificant correlations (*p* > 0.05) are omitted. Abbreviations are explained in the Methods section.

In the global context of biodiversity research through citizen science, socioeconomic factors play a lesser role than a country's biodiversity value. Figure 3 shows that only inbound tourism and land area, which can also be indirectly related to biodiversity values, exhibit a moderate correlation (0.5 < *r* ≤ 0.7) with the number of identifiers, observers, observations, and species recorded on iNaturalist. In contrast, socio‐economic factors such as population size, the Human Development Index, life expectancy, years of schooling, and GNI per capita show no significant impact on the development of citizen science (*r* < 0.5). At the same time, biodiversity indicators such as the number of endemic species, total species richness, and species with low occurrences exhibit the strongest correlations (*r* > 0.7) with the number of general observations, as well as with the number of identifiers on iNaturalist. Notably, among all socioeconomic and biodiversity factors, only the number of species with low occurrences and the total countries' species moderately correlates (0.5 < *r* ≤ 0.7) with the number of users and observations on eBird. No correlations are observed between Observation.org occurrences on GBIF and any socioeconomic or biodiversity factors, which may be attributed to the platform's varying popularity across different countries.

**FIGURE 4:**
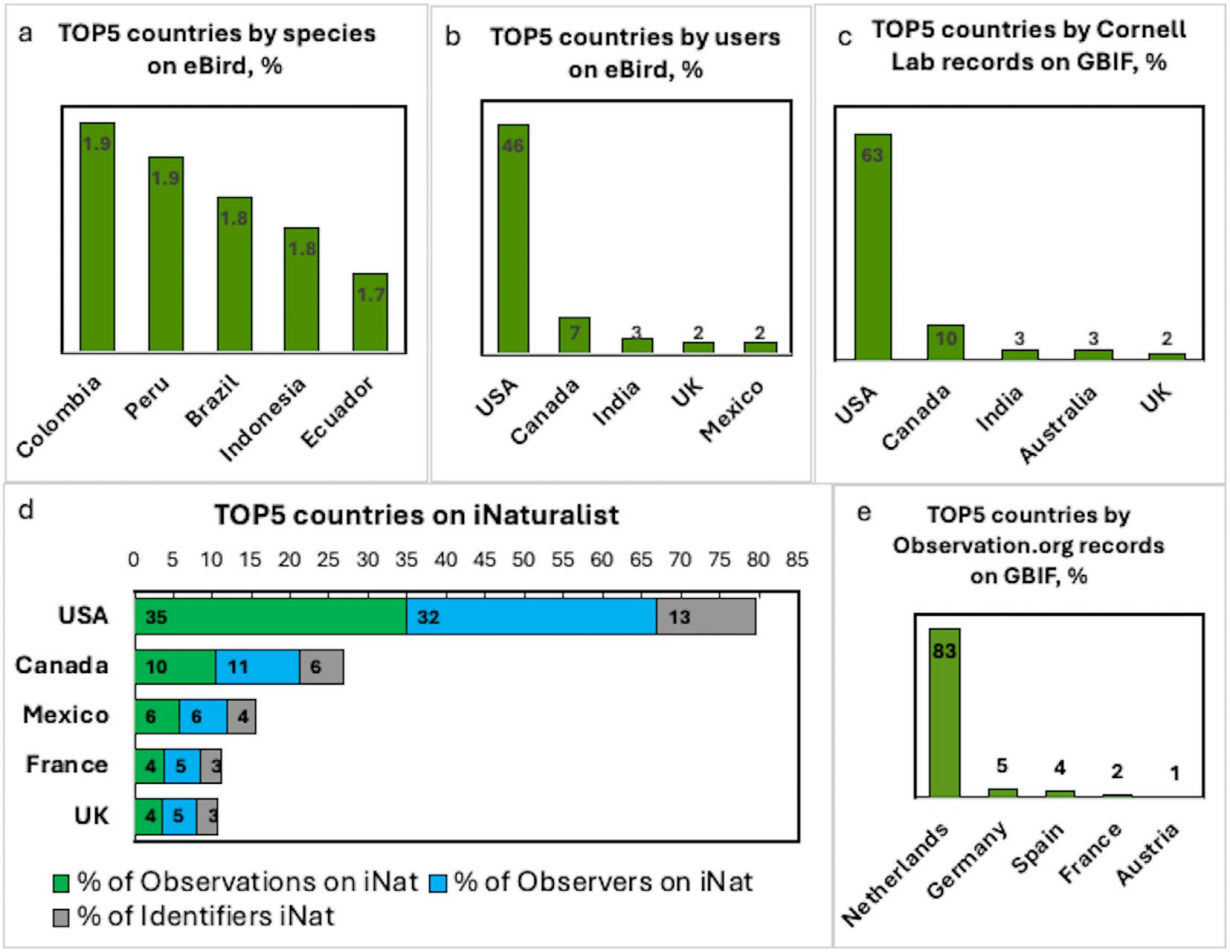
Top 5 countries on eBird by number of species (a); by eBird users and observations on GBIF (b, c); on iNaturalist by observations, observers, and identifiers (d); and on Observation.org by observations (e) (as a percentage relative to all 193 countries).

Therefore, natural factors such as land area and high, unique biodiversity may explain the greater number of recorded species on iNaturalist and eBird (Figure 3). This is further supported by an analysis of the top five countries by species count on eBird, which shows the highest numbers were recorded in Colombia, followed by Peru, Brazil, Indonesia, and Ecuador—all large, biodiverse countries that encompass globally recognized biodiversity hotspots (Myers 1990; Mittermeier et al. 2011) (Figure 4). In contrast, socioeconomic factors may influence the number of observers and identifiers. The top countries by users and occurrences on eBird, as well as by observations, observers, and identifiers on iNaturalist, are the USA and Canada, countries with large areas and high socioeconomic development. Other leading countries on eBird by users include India, the UK, and Mexico, while for occurrences, India, Australia, and the UK. On iNaturalist, Mexico, France, and the UK are the other top countries across all three indicators. More than 80% of Observation.org records originate from the Netherlands, where the platform was founded and is hosted. Other leading contributors include Germany, Spain, France, and Austria, all of which are EU countries. Interestingly, a similar pattern is observed with eBird and iNaturalist, where the top contributing country is the U.S., the country where these platforms were founded.

**FIGURE 5:**
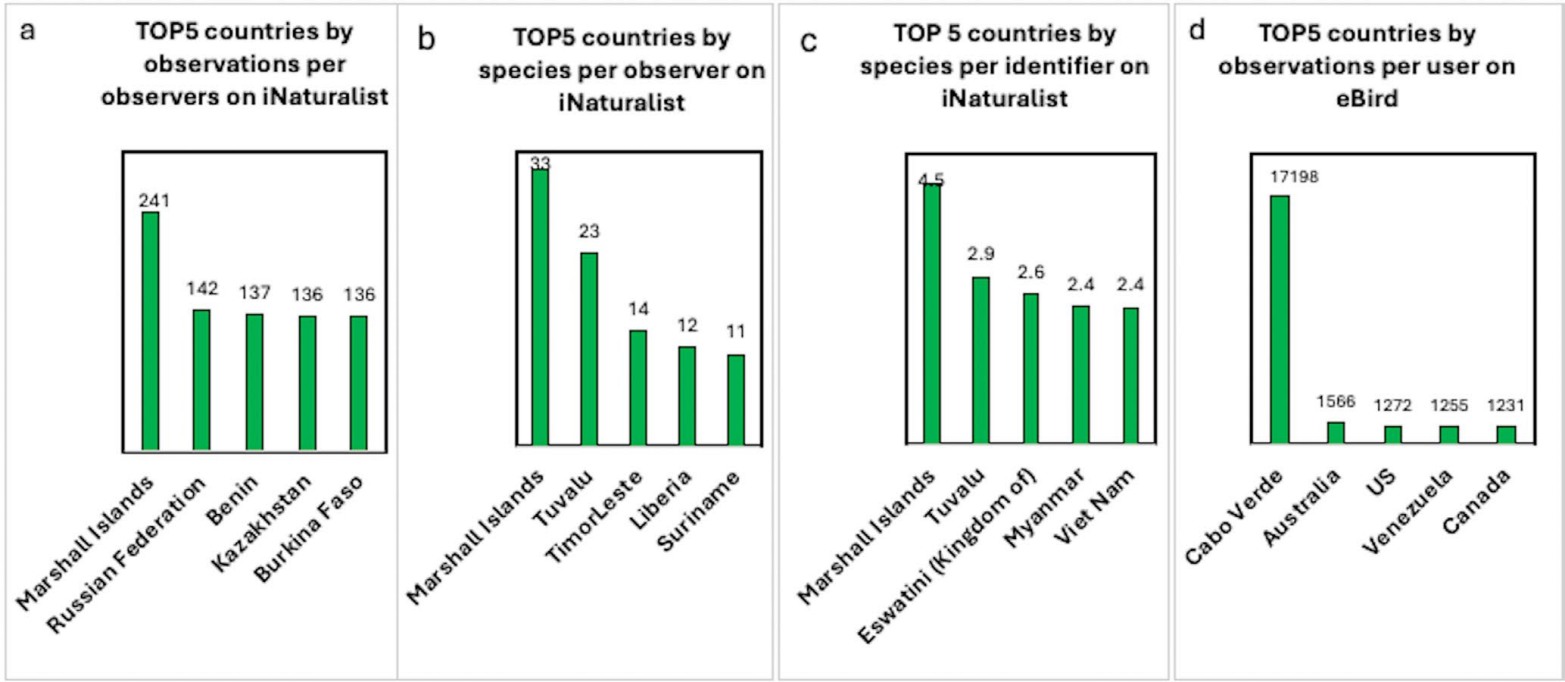
Top 5 countries on iNaturalist by observations per observer (a), species per observer (b), and species per identifier (c), as well as on eBird by observations per user (d).

Another important aspect is the relationship between the number of users (observers and identifiers) and the number of species and occurrences. As shown in Figure 3, strong correlations (*r* > 0.7) are observed between iNaturalist users (both identifiers and observers) and the number of observations and species. In contrast, the pattern on eBird differs. While there is a strong correlation (*r* > 0.7) between the number of users and total occurrences, the correlation between users and species is weak (*r* < 0.5). While this relationship will be explored in more detail, initial findings highlight notable differences in user contribution. Figure 5 shows that the top five countries on iNaturalist by observations per observer are the Marshall Islands, Russian Federation, Benin, Kazakhstan, and Burkina Faso—distinct from the top five countries by total number of observations (Figure 4). For instance, although the United States has the highest total number of observations, it averages only about 58 observations per user. In contrast, the Marshall Islands rank 144th in total observations, yet they record approximately 241 observations per observer. A similar pattern is observed in the rankings by number of species per observer and species per identifier, where the Marshall Islands again lead. It is important to note that the Marshall Islands are part of the Polynesian–Micronesian biodiversity hotspot (Myers 1990; Mittermeier et al. 2011), which may provide greater opportunities for recording a higher diversity of species and observations, but this is only one of several possible explanations. Another possible explanation is the presence of users with many observations, which may reflect the efficiency of individual contributors. This pattern may be consistent with the Pareto principle, which suggests that 80% of outcomes can result from 20% of cases. According to a recent study (Chozas et al. 2023), this pattern may also be influenced by engagement efforts that encourage higher levels of participation among contributors. The individual efficiency may also be driven by a greater interest in the rich and unique biodiversity characteristic of the country's leading in this indicator. The results concerning the number of species per observer on iNaturalist are also interesting (Figure 5c). Notably, the Marshall Islands, Tuvalu, Eswatini, Myanmar, and Vietnam rank highest for this indicator, making them particularly interesting from both biodiversity and political perspectives. Importantly, physical presence is not required to identify species; therefore, the motivation and research interests of individual identifiers likely play a decisive role in shaping this pattern.

Analysis of the top countries on eBird by observations per user reveals that Cabo Verde is the clear leader, with 17,198 observations per user. In second place is Australia, with 1566 observations per user, followed by the United States (1272), Venezuela (1255), and Canada (1231). Although Cabo Verde does not rank among the top five countries by number of users, species, or total observations on eBird (Figure 4), its lead in observations per user may be explained by its location within the Mediterranean Basin biodiversity hotspot (Myers 1990; Mittermeier et al. 2011) and the increasing popularity of birdwatching tourism in the country (Archipelago Choice 2025; Fatbirder 2025).

### 3.2 Examples of Citizen Science Engagement in Biodiversity‐Related Research Projects

#### 3.2.1 Case Study 1: Citizen Science in Marmot and Hamster Localities Research in Sumy and Poltava Regions, Ukraine

In 2024, citizen science involvement in marmot and hamster localities research in Sumy and Poltava Regions, Ukraine, led to the identification of 95 *Marmota bobak* localities and 3 *Cricetus cricetus* localities (Table 1). Of these, 40 marmot localities and all 3 hamster localities were newly recorded and had not been previously documented. It exceeds the previously documented numbers of 34 and 2 localities, respectively. Most localities (43 and 1, respectively) were reported through personal conversations, primarily during fieldwork. Citizen scientists were also effectively engaged through local Facebook groups and amateur communities, such as the Romny Division of the Ukrainian Society of Hunters and Fishermen, while official Facebook pages and print or online media proved less useful. Most respondents used the same communication channel through which they learned about the data collection effort, primarily by commenting on the original Facebook post or sending private messages via Messenger. However, seven messages were received through other channels, making it difficult to identify which post they were responding to. These reported localities have been categorized as “Other.”

Personal communication proved the most effective way to gather information, likely due to trust in local researchers and the researcher's student status, which encouraged participation. Respondents were generally open and eager to share knowledge, often using local toponyms, highlighting the need to account for naming variations. Mobile mapping tools were rarely used, and many could not provide coordinates or screenshots. Popular citizen science platforms like iNaturalist were also less effective for recording species locations (Table 1).

#### 3.2.2 Case Study 2: Citizen Science Contributions to Non‐Native Pink Salmon Research in Finland

The pink salmon citizen science campaign led to the measurements of 188 individual carcass habitats (Table 2), which accounted for 75% of all measured habitats during the research campaign. The researchers' prior knowledge, based on which the additional 60 carcass habitats were measured, was mostly based on citizen knowledge received in previous years through personal communications with the local people.

In this case study, the Finnish citizen science platform LAJI.FI (2025) and the international platform iNaturalist (2025) were of limited use. While they provided a general idea of where the species had been observed, the records typically referred to live individuals, whereas the research campaign focused on carcasses. When comparing the two platforms, the national platform Laji.fi yielded more observations than the international iNaturalist. Text messages seemed to be the preferred communication method, while social media appeared to be less significant, indicating that print media ads reached a wider audience than social media. Text messages were likely preferred for the anonymity they offered over public forums. Citizen carcass reports proved to be an effective tool in locating carcass sites and thus enabling targeted habitat measurements. Moreover, many of the messages were sent by people who noticed carcasses in the yards of their private shore cabins and guaranteed permission for the researchers to visit these cabin yards. These sites and the habitats they contain would have been unreachable without the citizen science component.

Citizen science greatly enhanced campaign efficiency by providing accurate, near‐real‐time location data that enabled researchers to concentrate on confirmed carcass sites, although vague or missing habitat descriptions often required site visits.

#### 3.2.3 Case Study 3: Citizen Science in Plant Species Research in the Dnipropetrovsk Region, Ukraine

The involvement of citizen science in floristic research in the Dnipropetrovsk Region, Ukraine (Table 3), has contributed to identifying six localities of *Astragalus ponticus* , one locality each for *Adonis vernalis* and *Gladiolus tenuis*, all species listed in the Red Data Book of Ukraine (2021). Among regionally rare plants, citizen scientists reported five localities of *Ephedra distachya* and one for *Onosma tinctoria*. However, no localities were reported by citizen scientists for *Anacamptis morio*, a species included in the Red Data Book of Ukraine, nor for two regionally rare species: *Asplenium trichomanes* and *Sempervivum ruthenicum*.

Regarding narrowly distributed species, citizen scientists recorded three localities for *Dianthus trifasciculatus*, two for *Jurinea cyanoides*, and one locality each for *Dianthus carbonatus* and *Centaurium erythraea* .

The highest number of recorded localities related to alien and invasive species, particularly *Asclepias syriaca* (17 localities) and *Grindelia squarrosa* (5 localities). This differentiation in recorded occurrences may be attributed to the highly invasive nature of *Asclepias syriaca* and *Grindelia squarrosa* . In contrast, *Datura* spp. is an alien species with a less frequent occurrence.

The most effective ways of engaging citizen scientists in this research were personal communication and Facebook. However, the number of localities recorded for the targeted species on iNaturalist was higher than those reported through other channels.

The results of this study indicate that the frequency of species occurrence significantly influences the number of detected localities. Citizen scientists did not report any localities for 1 out of 4 species listed in the Red Book of Ukraine and 2 out of 4 regionally rare species. Engaging citizen scientists through personal contacts, followed by expert verification by a botanist, is the most effective approach for rare, poorly studied species or those with difficulty in identification. In contrast, widespread species, without any identification issues, are best monitored through observations recorded on citizen science platforms and social networks. Monitoring invasive species can also be effective using citizen science platforms, but this depends on the difficulty of their identification and on the presence of similar species in the studied area. In our case, monitoring with iNatrulist was most effective for *Asclepias syriaca* , whereas it was less effective for *Datura* spp. and *Grindelia squarrosa* . The reason for this may be that *Datura* spp. and *Grindelia squarrosa* are not very widespread, and there may be difficulties in their identification. Whereas the *Asclepias syriaca* is very widespread and it is difficult to confuse with other species.

## 4 Discussion

An analysis of data contributors to GBIF reveals that a significant proportion of records now originate from citizen science platforms, increasing from approximately 20% in 2001 to over 90% in 2023, primarily from eBird, iNaturalist, and Observation.org. Moreover, among the top publishers on GBIF, the most frequently cited datasets are those from iNaturalist.org and Observation.org, both of which are fully citizen science platforms. The differences in citation rates may be caused by unequal data collection for all taxonomic groups by citizen science platforms. In particular, eBird collects data only on birds, while iNaturalist and Observation.org cover all taxonomic groups. While these platforms demonstrate high contribution in data volume and citation impact, concerns remain regarding data quality and consistency. For example, on iNaturalist, a key challenge is the low quality of images submitted with observations, which often obscures accurate species identification and can lead to misidentifications, even among observations marked with Research Grade status (White et al. 2023; López‐Guillén et al. 2024; Alfeus et al. 2025). Incorrect species identification can lead to errors in research literature and inaccurate conclusions. Despite these quality challenges, several effective ways exist to improve data quality on platforms like iNaturalist, especially through clear and effective communication. These include raising awareness, improving understanding of common identification errors and the species identification process, providing stronger mentorship and coordination, especially for new users, etc. (McMullin and Allen 2022; Johnston et al. 2023; White et al. 2023; López‐Guillén et al. 2024).

Another key factor in improving data quality is understanding what motivates citizen scientists. Gaining insight into citizen scientists' motivations can help better understand their behavior and develop more effective strategies for engagement and contribution. The most influential motivational themes include contributing to science and conservation, the desire to gain new knowledge and skills, spending time outdoors, connecting with nature, and engaging with a like‐minded community (Beza et al. 2017; West et al. 2021; Agnello et al. 2022; Thompson et al. 2023; Kozak et al. 2024). Our data show that globally, natural factors like biodiversity and hotspot location influence citizen science biodiversity data more than socio‐economic conditions of the countries. Our assumption is that countries with unique biodiversity and large or ecologically diverse territories tend to attract more tourists, amateur naturalists, and researchers, which can also contribute to participants' motivation. This interpretation is supported by our findings on trends in the number of observations per user. The presence of interesting biodiversity may encourage individual observers to accumulate more records, although the observed trends may also reflect the effects of platform promotion and engagement efforts. A study in Hawaii supports this statement, revealing that enthusiastic visitors were the main contributors to iNaturalist, while residents were the minority, and their observations were more likely to be casual, with a focus on human‐impacted landscapes (Dimson and Gillespie 2023). Raising awareness of citizen science tools like iNaturalist among tourists, in partnership with tour operators, could be an effective strategy for promoting engagement and data collection. Another important issue is the motivation and engagement of qualified specialists in identifying observations. Records that remain unidentified cannot achieve Research Grade status and, consequently, cannot be used in further scientific analyses. Because physical presence is not required for species identification, experts can contribute from anywhere in the world. However, their motivation is likely influenced by factors such as research interests, the quality of the submitted observations, and the uniqueness or scientific value of the records.

Finally, it should be noted that this study relies solely on correlation analysis, which has inherent limitations. In particular, factors such as the country of origin of a platform and patterns of its subsequent use may substantially influence the strength of correlations with socio‐ecological factors. For example, even the geographic distance from a platform's country of origin may affect the observed relationships. Therefore, more detailed statistical analysis in future studies, for example, multivariate linear regression, will help explore potential nonlinear effects or multivariate interactions which help to understand these relationships deeper.

The three case studies in this publication demonstrate that often the most effective way to engage citizen scientists is through direct, personal communication and tailored requests shared via social media and other channels. While citizen science platforms like iNaturalist can be valuable, their contribution increases when combined with active communication across multiple channels like social media. Combining citizen science platforms with social media can be an effective strategy for enhancing citizen science engagement, facilitating user interaction and coordination, raising awareness, enabling the formation of online volunteer communities, and offering a range of other benefits (Oliveira et al. 2021; Spasiano et al. 2021). It is important to recognize that training volunteers is a long‐term process, and social networks can support ongoing learning, ultimately helping to improve both the quality and quantity of collected data.

Face‐to‐face interactions are especially effective for engaging citizen scientists with rare and/or difficulty in identifications species, particularly in areas with limited digital access or trust in technology. Expert verification should follow personal engagement for accuracy. In contrast, widespread, without any identification issues, are better monitored through digital platforms like iNaturalist, supported by outreach via social media. Citizen science can also be an effective tool for detecting and monitoring invasive species (Pocock et al. 2024), but it should consider their specificity and possible difficulties with identification.

## 5 Conclusions

Citizen science platforms have become the dominant source of biodiversity data on GBIF. The global contribution of citizen science in generating biodiversity data is shaped more by a country's ecological uniqueness (like unique biodiversity and location within biodiversity hotspots) than by its economic status, social development, or population size. Nations with large or biologically diverse territories tend to attract more engagement from tourists, naturalists, and researchers, leading to broader observation coverage. However, high user numbers or total observations alone do not equate to greater contribution. Overall, the findings illustrate that biodiversity value, platform origin, and user participation all shape the global landscape of citizen science contributions to GBIF.

The best way to monitor rare, poorly studied, or species with difficulty in identification is through personal contact with citizen scientists, followed by expert verification. On the other hand, widespread species, without any identification issues, are more effectively tracked using citizen science platforms like iNaturalist, along with support from social media outreach such as Facebook.

Therefore, a mixed approach that leverages various channels of citizen science communication and engagement, combining global biodiversity platforms like iNaturalist with social media and, in some cases, personal communication, can be an effective strategy for increasing both the volume and quality of contributed data.

## Author Contributions

**Olena Kozak:** conceptualization (equal), data curation (equal), formal analysis (equal), methodology (equal), resources (equal), visualization (equal), writing – original draft (equal), writing – review and editing (equal). **Aino Erkinaro:** investigation (equal), methodology (equal), resources (equal), writing – original draft (equal), writing – review and editing (equal). **Kaisa‐Leena Huttunen:** investigation (equal), methodology (equal), resources (equal), writing – original draft (equal), writing – review and editing (equal). **Larysa Osypenko:** investigation (equal), methodology (equal), resources (equal), writing – review and editing (equal). **Svitlana Kyiak:** investigation (equal), methodology (equal), resources (equal), writing – review and editing (equal). **Nataliia Matsai:** investigation (equal), writing – review and editing (equal). **Mykyta Peregrym:** conceptualization (equal), data curation (equal), methodology (equal), writing – original draft (equal), writing – review and editing (equal).

## Funding

This work was supported by Research Council of Finland (336449, 356403, 359684) and Kvantum‐Instituutti, Oulun Yliopisto.

## Conflicts of Interest

The authors declare no conflicts of interest.

## Supporting information


**Table S1:** ece373461‐sup‐0001‐TableS1.docx.

## Data Availability

All data supporting the findings of this publication are available within the text and in Supporting Information. All supplementary files are available via the link—https://github.com/Olene/CitSci‐in‐World.
