# Supplementary material for: Effective Data Collection Approaches for Citizen Science in Biodiversity Research
Source: Ecol Evol. 2026 Apr 8;16(4):e73461. doi: 10.1002/ece3.73461 (PMC13062650; doi:10.1002/ece3.73461)
Supplement: Supplementary file 1 — Table S1: ece373461‐sup‐0001‐TableS1.docx. [file ECE3-16-e73461-s001.docx]

**Supplementary Table 1.** Information dissemination channels for citizen scientist involvement in three case studies.

| **Description** | **Weblink** |
| --- | --- |
| *Case Study 1: Citizen Science in Marmot and Hamster Localities Research in Sumy and Poltava Regions, Ukraine* | |
| Official Facebook page of the local newspaper “Visti Romenshchyny” where the questionnaire on marmot and hamster localities was distributed. | <https://www.facebook.com/vistiromen> |
| Post on the official Facebook page of the local newspaper “Tandem” encouraging reports of marmot and hamster localities (in Ukrainian). | <https://www.facebook.com/tandem.romny/posts/pfbid0mjjAZ7opi4xg6XoT88BcPudkSp68JqGYpgXbg6c5mukz5PCBtGKRJMrKQL3XC5LWl> |
| Publication on the official Lubny City Council website encouraging reports of marmot and hamster localities (in Ukrainian). | <https://lubnyrada.gov.ua/oholoshennia/10287-shukayemo-khomyaka-i-babaka-na-sumshchyni-ta-poltavshchyni> |
| Post on the official Facebook page of Luhansk Taras Shevchenko National University encouraging reports of marmot and hamster localities (in Ukrainian). | <https://www.facebook.com/LNU2022/posts/pfbid0uQecfYxZ3ggCGKSzY6LBvDVwDUmFTTQLU4edvcZgmvYJwPWuxq3o1QCL9jvA8wM6l> |
| *Case Study 2: Citizen Science Contributions to Non-Native Pink Salmon Research in Finland* | |
| Publication on the official University of Oulu website encouraging public assistance in reporting pink salmon carcass locations (in English). | <https://www.oulu.fi/en/news/how-will-dead-pink-salmon-affect-northern-nature-researchers-are-asking-public-for-help> |
| Publication on the official University of Oulu website encouraging public assistance in reporting pink salmon carcass locations (in Finnish). | <https://www.oulu.fi/fi/uutiset/kyttyralohien-raadoista-vapautuvat-ravinteet-voivat-muuttaa-pohjoista-luontoa-kansalaishavaintoja> |
| Publication in newspapers “YLE” highlighting the research and promoting citizen science participation (in Finnish). | <https://yle.fi/a/74-20042859> |
| Publication in newspapers “METSASTYS JA KALASTUS” highlighting the research and promoting citizen science participation (in Finnish). | <https://metsastysjakalastus.fi/loysitko-kuolleita-kyttyralohia-ilmoita-havaintosi-tutkijoille/> |
| Publication in newspapers “MAASEUDUN TULEVAISUUS” highlighting the research and promoting citizen science participation (in Finnish). | <https://www.maaseuduntulevaisuus.fi/metsa/49a7fa99-0215-4693-a32f-d84d33b12047> |
| Publication in newspapers “Apaa-Ajan Kalastajalehti” highlighting the research and promoting citizen science participation (in Finnish). | <https://www.vapaa-ajankalastajalehti.fi/uutiset/kyttyralohiraatojenkertymapaikoistakerataantietoa130923/> |
| Article in newspapers “The Quardian” highlighting the research and promoting citizen science participation (in English). | <https://www.theguardian.com/environment/2023/oct/02/it-smells-so-bad-glut-of-wild-salmon-creates-stink-in-norway-and-finland> |
| Post in Facebook to involve citizen science. | <https://www.facebook.com/groups/5422278262/permalink/10159799383118263/> |
| Post in X about reporting the locations of pink salmon carcasses. | <https://x.com/AinoErkinaro/status/1684886833585356801> |
